# Performance, blood and antioxidant status in dual-purpose laying hens supplemented with aqueous extract of Christ’s thorn jujube (*Ziziphus spina-christi* L.) leaves as phytogenic agent in subtropical conditions

**DOI:** 10.5713/ab.23.0243

**Published:** 2024-01-14

**Authors:** Khaled H. El-Kholy, Hasan Tag El-Din, Found A. Tawfeek, Vincenzo Tufarelli, Caterina Losacco, Rashed A. Alhotan, Manal E. Shafi, Mohamed A. Korish, Youssef A. Attia, Sara H. M. Hassab

**Affiliations:** 1 Animal, Poultry and Fish Production Department, Faculty of Agriculture, Damietta University, Damietta 34511, Egypt; 2 Agricultural Research Center, Giza, Giza Governorate 12618, Egypt; 3 Department of Precision and Regenerative Medicine and Jonian Area (DiMePRe-J), Section of Veterinary Science and Animal Production, University of Bari ‘Aldo Moro’, Bari 70010, Italy; 4 Department of Animal Production, College of Food and Agricultural Sciences, King Saud University, Riyadh 11451, Saudi Arabia; 5 Department of Biological Sciences, Zoology, Faculty of Sciences, King Abdulaziz University, Jeddah 21589, Saudi Arabia; 6 Agriculture Department, Faculty of Environmental Sciences, King Abdulaziz University, Jeddah 21589, Saudi Arabia; 7 Animal and Poultry Production Department, Faculty of Agriculture, Damanhour University, Damanhour 22713, Egypt

**Keywords:** Antioxidant Status, Christ’s Thorn Aqueous Extract, Laying Hens, Performance

## Abstract

**Objective:**

The potential of aqueous extract of Christ’s thorn jujube (*Ziziphus spina-christi*) leaves (SLAE) to reduce the negative impacts of heat stress on production performance and physiological traits was investigated in dual-purpose layers under subtropical farming.

**Methods:**

A total of 200, 25-week-old laying hens (Inshas strain) were randomly assigned to four dietary treatments including SLAE at 0, 2.5, 5.0, and 7.5 mL/kg, respectively. The average temperature-humidity index value was 26.69 during the experimental period. The SLAE contained saponin (0.045%), total flavonoid content of 17.9 mg of quercetin equivalent/ 100 g and overall antioxidant capacity concentration of 17.9 mg of ascorbic acid equivalent/ 100 g.

**Results:**

The maximum final body weight (BW), BW gain, egg weight, number, and mass occurred at the level of SLAE7.5 inclusion. The egg quality was significantly higher in SLAE groups than in control, and overall, SLAE7.5 had the most favorable influence at 28 and 32 weeks. Liver and kidney function, as well as lipids profile, improved significantly by SLAE inclusion; the lowest concentrations of these parameters were in SLAE7.5 hens. Treatment with SLAE7.5 increased total antioxidant capacity and endogenous antioxidant enzymes compared to control, whereas no effect on superoxide dismutase was noticed.

**Conclusion:**

The addition of SLAE at 7.5 mL/kg diet improved egg laying performance and quality, metabolic profiles, and antioxidant status during hyperthermia conditions.

## INTRODUCTION

Climate change is a major challenge to poultry production especially in tropical and sub-tropical areas [1]. According to the scientific community, the temperature of the planet surface has increased over the years, causing climate changes, this situation adversely affected livestock production globally. Poultry, particularly layers, are one of the livestock species that are particularly vulnerable to climate change [2]; a number of laying hens farms are continuously growing to cover the high demand for animal products such as eggs. However, like other poultry species, laying hens are very sensitive to fluctuations of ambient temperature (AT). Some researchers have studied the effect of climate on productivity, product quality, and welfare of laying hens and found decreased productivity and increased mortality as an effect of heat stress that was as-sociated with significant economic losses for farmers of laying hens [3].

The lowered performance and survivability of laying breeders caused by heat stress is usually observed in tropical and subtropical area conditions with substantial losses for laying hens farming [3]. Heat stress is also a severe management issue that threatens the antioxidant status of the organism, as reflected by elevated levels of oxidative stress and lipid peroxidation and decreased antioxidant plasma concentrations [4]. These factors consequently impair the health and safety status of poultry and adversely influence production parameters such as live weight, feed efficiency, egg production, egg quality, eggshell quality, fertility, hatchability, and survival [5]. Oxidation status is, therefore, an index that should be considered in the poultry industry and research. Interest in using phytogenic substances and botanical materials is increasing because these substances are considered safe and environmentally friendly in addition to being effective in improving laying performance and egg quality due to their beneficial bioactive compounds [6]. The majority of these phytochemicals (plant-derived chemical substances) possess strong chemopreventive and antioxidant properties [7]. Consumer demand for food items that are free of dangerous residual compounds can be met if natural antioxidants replace synthetic compounds, leading to great benefits for the poultry sector [8]. This novel concept was based on the idea that supplying different natural antioxidants to birds could help them cope with stress more successfully [9].

*Ziziphus spina-christi* is a subtropical plant known in Egypt as ‘Nabq’ or ‘Seder’ and in English as Dom/Christ thorn. It belongs to the genus *Ziziphus* under the family *Rhamnaceae* [10]. This genus contains over 100 species of deciduous and evergreen trees and shrubs that grow in tropical and subtropical climates [11]. The local Arabs use all components of the plant to assist them in maintaining a healthy lifestyle; hence the species Zizyphus has a great medicinal significance [12]. Phytochemically, *Zizyphus spina-christi* L. is known for its cyclopeptide alkaloids and polysaccharides [13]. At the same time, several flavonoids, tannins, and saponins have been identified in different species of this important genus [14]. Recently, many researchers have examined the plant extracts of Ziziphus species and found that they possess antibacterial [15] as well as antifungal [16], anticancer [17], and antioxidant [18] activities. *Z. spina-christi* has been shown to protect Albino Wistar rats from carbon tetrachloride (CCL4)-induced oxidative stress and hepatotoxicity [19]. The antioxidant and free radical scavenging properties of *Ziziphus spina-christi* leaves (SLAE) may also play a role in hepatoprotection. Isolated flavonoids from the SLAE [20]. Plant phenolics are well known for their ability to scavenge free radicals and hence act as antioxidants [18]. Phytogenic feed additives (PFA) may contain polyphenols, organic acids, essential oils, terpenoids, and aldehydes, which have been demonstrated to boost laying performance and egg quality in laying hens [21]. Thus, the phytochemicals found in SLAE may be responsible for their *in vitro* antioxidant effects. Nowadays, there is an urgent need to explore promising alternatives belonging to natural antioxidants that could increase poultry productivity under heat-stress conditions.

Therefore, this study aimed to assess the *in vivo* antioxidant properties of leaf extracts of *Z. spina-christi* grown in Egypt under subtropical conditions and to quantify the total phenolic content in aqueous extract. This study will open new areas of application of an aqueous extract of this plant as an antioxidant agent in dual-purpose laying hens reared under subtropical conditions.

## MATERIALS AND METHODS

The experiment protocol was carried out in accordance with the general guidelines of the Declaration of Helsinki and approved by the Animal Care Ethics Committee of Animal Production Research Institute, ARDC, Giza, Egypt, under protocol No. 429-03-05-01/2019.

### Preparation of *Z. spina-christi* extract

Fresh leaves of *Z. spina-christi* at flowering stage were collected from different locations in New Valley, Egypt, during the spring of 2019. The collected plant material was rinsed with distilled water to remove any dust and particulate matter and dried at room temperature for several days. Dried leaves (~100 g) were ground and extracted in one liter of distilled water for 24 h. The preparation was then filtered through Whatman No. 4 with squeezing by hand to separate the debris from the filtrate (250 mm filter papers), and the SLAE extract was placed in clean containers and used fresh. This procedure was carried out weekly, and the filtrate was incorporated into experimental diets. For further analysis, the SLAE extract was stored at −20°C [22]. The combination treatments were prepared in the layer hens’ house before adding to the diet.

### Identified compounds in aqueous extract of SLAE

The three samples of SLAE were analyzed using gas chromatography-mass spectrometry (GC-MS), Thermo Scientific ISQ LT Trace 1310, to identify its chemical constituents. The injector temperature was 250°C, and the column was described as follows: TG-SQC GC Column 15×0.25×0.25 mm, the temperature was programmed to rise steadily from 50°C to 290°C as follows: The oven temperature began at 50°C and gradually raised to 150°C at a rate of °C/min (held for 1 minute), 250°C at a rate of 5°C/min (held for 5 minutes), and ultimately 290°C at a rate of 10°C/min (held for 2 minutes). The MS transfer line temperatures and ion source were 300°C. A spitless mode was used to inject the crudes. At 70 eV, mass spectra of pieces ranging from 40 to 1,000 D were obtained. The mass spectra of peaks were computer matched with the Wiley and National Institute of Standards and Technology (NIST) libraries’ mass spectral database for final confirmation of constituents.

Using standard procedures, the SLAE samples were submitted to qualitative phytochemical analysis for the presence of tannins, saponins, glycosides, flavonoids, alkaloids, terpenes, steroids, and other active chemical elements [23,24]. Total antioxidant activity is determined using the phosphormolybdenum method [25]. The total flavonoids content was measured using the Dowd method, which was developed from the aluminum chloride colorimetric approach [26].

### Animals and experimental design

This research work was carried out at the Animal Research Station, Sakha Agricultural Research Center, Egypt, during the period from September to November. A total of 200, 25-week-old laying hens (Inshas strain) were used, and the trial lasted 12 weeks. Hens were randomly assigned to 4 groups: 0 (control), 2.5, 5.0, and 7.5 mL/kg SLAE added in diets, respectively. There were five replications per group (10 hens). Replicates were randomly housed in separate rooms (260×210 cm) on a littered floor. All birds were raised under the same management, hygienic, and environmental settings. During the experiment, the feed was supplied *ad libitum*, and the light schedule was 16 h of light and 8 h of darkness every day. Table 1 shows the ingredient composition and nutrient content of basal diet. Diets were formulated to exceed or meet the nutrient requirements of laying hens [27]. The composition of the experimental diet was determined according to the AOAC [28].

### Environmental variables

Average AT (°C) and relative humidity (RH, %) inside the building were determined weekly using a standard thermometer (Brannan, China) with a 42°C calibration and a standard hygrometer (Cheemi, China) with a 50°C calibration respectively. Then, the temperature-humidity index (THI, units) was calculated using the equation by of Zulovich and DeShazer [29] as follows: THItb = 0.60Tdb+0.40 Twb; where: THItb = temperature humidity index for laying hens; Twb = wet bulb temperature (°C) Tdb = dry bulb temperature (°C); where: db °C = dry bulb temperature in Celsius, RH = relative humidity percentage/100. According to THI values, the environmental conditions were categorized as: no heat stress (<27.8), moderate heat stress (28.8 to 28.8), severe heat stress (28.9 to 29.9), and very severe heat stress (>30.0).

### Laying performance and egg quality

Live body weight (BW, g) was recorded at 24, 28, and 36 weeks of age. Throughout the period, feed intake (FI, g) was recorded weekly for each replicate per group, and then body weight gain (BWG, g) of hens was calculated by the difference between final and initial weight. Individual egg weight (EW, g) and egg number (EN) were recorded daily, then the average egg mass (EM) percentage was calculated for each replicate and group. Egg mass (g/g) was calculated by multiplying the EN by EW. For each group, the feed conversion rate (FCR) was calculated weekly as kg of feed consumed per kg of eggs produced. Every Saturday, six eggs were collected at random from each replication to assess egg quality during the trial. Egg quality analysis was performed at 28 and 32 weeks of age. Separate weights were measured for the yolk and albumen. The yolk index (%) was calculated using the equation (yolk height/yolk diameter) ×100. The color of egg yolk was determined by comparing it to one of the Roche yolk color fans having 15 bands. Haugh units, which are the height of egg albumen broken off on a flat surface, were measured using a tripod micrometer.

### Serum biochemistry

Blood samples (~5 mL) were taken from the wing vein of 10 hens per experimental group at the end of the experiment (36 weeks of age) and collected into a tube containing 1% ethylenediaminetetraacetic acid Blood was layered on 1077 Histopaque (Sigma, 10771; Sigma-Aldrich, St. Louis, MO, USA) and centrifuged at 4°C for 15 min at 3,000 *g*, and serum samples were kept at 4°C for 2 days until analysis. Individual samples were analyzed for serum total protein and albumin by using a commercial kit according to guidelines [30,31], respectively. Albumin measurements were subtracted from total protein data to determine globulin levels. Serum samples were also analyzed for concentrations of aspartate (AST, U/L) and alanine amino transaminases (ALT, U/L) using commercial kits (Linear Chemicals, Barcelona, Spain) according to the manufacturer’s procedure. Also, the serum was assayed for total cholesterol (TC, mg/dL), total lipids (TL, mg/dL), triglycerides (TG, mg/dL), high-density lipoprotein (HDL, mg/dL), and low-density lipoprotein (LDL, mg/dL), creatinine (mg/dL) and urea (mg/dL) using standard protocol methods. A spectrophotometer UV4802 (Unico Co., Dayton, OH, USA) was used to quantify the activity of catalase (CAT, U/mg) calorimetrically, superoxide dismutase (SOD, U/mL), and serum malondialdehyde (MDA, nmol/mL) levels [32]. The procedures were conducted using an assay kit (Biosystem S.A, Costa Brava, 30, Barcelona, Spain).

### Statistical analysis

A MIXED procedure for repeated measurements (SAS, 2012, release 9.2, Cary, NC, USA) was used for assessing BW, BWG, FI, EN, EW, EM, external and internal egg quality, biochemical attributes, and redox status indicators as dependent variables. Levels of dietary SLAE, time of sampling and/or data collection (from week 25 to 36 for layer performance and external and internal egg quality variables and weeks 25 to 28, 29 to 32, 33 to 36, and 25 to 36 for biochemical variables) were introduced as fixed independent variables. Physiological variables were analyzed using a general linear model procedure (one-way analysis of variance; SAS, 2012). Differences among treatment groups were identified using Duncan’s new multiple ranges *post-hoc* test and were considered significant when p<0.05. All results were expressed as least square means± pooled standard error of the mean. Variance homogeneity and normality were examined by Shapiro-Wilk and Levene test. The level of significance was set at p<0.05.

## RESULTS

### Environmental variables

The mean values of AT, RH, and THI during the whole experimental period were 32.27°C±0.44°C, 71.65%±0.85%, and 30.54±0.43, respectively (Table 2). The highest THI value during the experimental period was recorded at the first experimental period (week 25 to 28), corresponding to the hottest period. On the other hand, the average THI value was 26.69 during the last experimental period (week 33 to 36).

### Chemical composition and phytochemical analysis of the extract

The aqueous extract contained 25 distinct chemicals, which accounted for 100% of the total extract. Table 2 listed the detected chemicals in order of their elution on the TG-SQC GC Column. Biotin (34.66%) and retinol (10.69%) were the most common chemicals found. As shown in Table 3, trans-13-Octadecenoic acid (0.61%), elemene (0.72%), and nobiletin (0.96%) were also included in the minor components. Table 4 shows the phytochemical analysis of an aqueous extract of SLAE, indicating the presence of the saponin (0.045%) and total flavonoid content (17.92 mg of quercetin equivalent [QE]/100 g). The same Table also illustrates the overall antioxidant capacity concentration (269.25 mg of ascorbic acid [AC]/100 g).

### Laying performance and egg quality

During the experimental period (25 to 36 weeks of age), both the welfare and general health status of birds were satisfactory. No signs of death and sickness or other deleterious influences were observed among groups. Significant improvements were observed in BWG and final BW; the greatest values were recorded in the group treated with 7.5 mL SLAE/kg diet compared with the control hens. The most efficient feed conversion ratio was found in the SLAE 7.5 followed by SLAE 5, and SLAE 2.5, under high AT. Considering the whole experimental period, FI in the supplemented groups was approximately similar. The control birds showed poorer performance and lower FI than the experimental groups (Table 5).

The effect of SLAE supplementation on the laying performance during the experimental period under hot environmental conditions is presented in Table 6. Interestingly, the incorporation of SLAE in diets during the whole period improved EN, EW, and EM significantly (p<0.05) compared with CON; the linear regression analysis of EW (p = 0.0100) and quadratic one for EN (p = 0.0058) and EM (p = 0.0029) showed that the greatest value was recorded at a level of 7.5 mL SLAE/kg diet. Regarding egg quality, the regression analysis showed that there was a quadratic relationship between dietary SLAE and egg albumen diameter (p = 0.0004), albumen index (p = 0.0056), yolk color (p = 0.0029), yolk index (p = 0.0013), and Haugh unit (p = 0.0004); the greatest values of albumen diameter, yolk color and Haugh unit (score) were observed at the level of 7.5 mL SLAE/kg diet, while the greatest values of albumen and yolk indices were recorded at a level of 5.0 mL SLAE/kg diet. Additionally, yolk diameter, height, weight, and egg albumen height increased linearly with increasing levels of SLAE.

### Serum biochemistry and redox index

The effects of dietary SLAE inclusion on serum biochemical constituents at the age 36 weeks are shown in Table 7. The SLAE inclusion significantly (p<0.05) affected all blood serum constituents at the end of the trial. Concentrations of TP and creatinine in blood serum tended to be affected by the treatment (p = 0.0848 and 0.0639, respectively); the greatest and lowest concentrations of TP and creatinine. Regarding lipid profile, blood serum concentration of TC, TL, and TG were affected linearly by treatments (Table 7); the lowest concentrations of these parameters were observed in the SLAE7.5 group. Higher levels of SLAE decreased linearly (p<0.01) ALT and AST concentration. Urea levels showed a significant linear response (p = 0.0157). However, the difference among the three levels of SLAE (2.5, 5.0, 7.5 mL/kg diet) was not significant for this parameter (Table 7).

Effects of the dietary SLAE inclusion on the redox status of layer hens are presented in Table 8. Interestingly, the regression analysis showed that there was a quadratic relationship between dietary SLAE and both TAC and CAT activity (p = 0.0001 and 0.0255, respectively); the greatest values were indicated at the levels of 5.0 and 7.5 mL SLAE/kg diet for TAC and CAT, respectively. The lowest MDA values were found in the SLAE7.5 group, whereas the highest value was recorded for glutathione in the same group compared to the control. In contrast, SLAE inclusion did not exhibit a significant effect on SOD levels (p = 0.6831).

## DISCUSSION

In tropical and subtropical areas, climate change has adverse effects on poultry performance during summer seasons and negatively affects the productivity of laying hens. Thus, eco-friendly production enhancers are essential to sustain performance and physiological status of animals. In the present study, the THI data indicated a severe heat stress condition (more than 30.0) (Table 2). The optimal THI for rearing layer hens has been proposed to be less than 27.8 [29]. Our data suggested that birds were subjected to heat stress as a result of both high AT and RH throughout the study period. This was also confirmed by the decline in layers output measures. Heat stress is also a severe management issue that threatens antioxidant status, as reflected by elevated levels of oxidative stress and lipid peroxidation and decreased antioxidant plasma concentrations [4].

One of the simplest ways to reduce heat stress, implicitly oxidative stress, is to use dietary addition with antioxidants from plants. As mentioned in Table 2, the major compounds detected in the chemical analysis of the aqueous extract of SLAE were biotin (23.51%) and retinol (10.69%), which have been previously associated with antioxidant activity [33]. These results agreed with previous phytochemical studies on different species of the genus Ziziphus except for the presence of alkaloids which are absent in the current study (aqueous extract) and ethanol extract [34]. Herbal flavonoids stimulate appetite in chickens and help maintain a healthy gut microbiota balance through their anti-inflammatory and antioxidant properties [35]. Besides that, Table 3 shows that the total antioxidant capacity and flavonoid concentration of SLAE were 17.92±0.09 mg of AC/100 g and 17.92±0.09 mg of QE2/100 g, respectively. The phytochemical examination of SALE revealed the presence of saponins, alkaloids, flavonoids, and tannins, which coincides with previous findings [36]. Ziziphus (*Rhamnaceae*) has been identified as a significant source of dietary antioxidants, particularly phenolic compounds [37]. According to the literature review, a range of cyclopeptides and isoquinoline alkaloids, flavonoids, terpenoids, and their glycosides were found in various levels in most *Ziziphus* species, particularly *Z. spina-christi*, having *in vitro* antioxidant properties [37]. The antioxidant activity and radical scavenging activities of flavonoids have been studied by many researchers [38,39].

The SLAE addition in hens diet resulted in greater final BW and BW gain than the control group. In general, the addition of herbal antioxidants to laying hens’ diet had no negative effect on production performance [40]. The BW gain is not a physiologically or commercially essential performance trait for laying hens; hence BW gain over the minimum levels is seldom pursued due to increasing maintenance energy requirements. Nonetheless, PFA’s beneficial effects on the usage of digestive products and the availability of key nutrients for absorption may increase BW gain within the product’s genetic potential without altering product performance. Due to the bioactive components found in SLAE, a beneficial influence of these phytogenic additions was observed as an improved BWG. The multi-biologically active components of SLAE, such as sterols, flavonoids, triterpenoids, sapogenins, and saponins, are effective in enhancing nutrient and energy metabolism as well as intestinal enzymes [41]. The ability of flavonoids to bind to extracellular and soluble proteins, as well as to bacterial cell walls, is the source of their activity [42], DNA, RNA, protein, and lipid production in bacteria are all inhibited [43], interfering with energy metabolism is akin to respiratory-inhibiting antibiotics because energy is required for active absorption of various metabolites and macromolecule synthesis [44]. The active components of SLAE display antibacterial, antioxidant, and digestive enzyme-stimulating effects [13]. As a result, these bio-active components could be employed to boost laying hen growth by promoting nutrient absorption and utilization as well as modulating beneficial microorganisms in the gastrointestinal tract. SLAE’s mode of action as PFA, however, is still unknown.

Results of FI showed that birds in the control group had the highest value (118.84 g/d), which is an indication that SLAE inclusion depressed FI, which might be be-cause of nutrient satisfaction. These findings agree with previous findings [45], who showed that when aqueous *Moringa oleifera* leaf extracts were administered to Hubbard broiler chicken, the control group had a greater FI than the SLAE treated groups. This could be due because of SLAE’s digestive and metabolism capabilities have improved, allowing it to meet nutrient requirements at lower FI. Flavonoids and phenolic compounds are abundant in SLAE [37]. This mixture is required for growth and decreases disease infestation in the gastrointestinal tract [46]; as a result, feed consumption will increase, perhaps reducing the need for bird care and production.

Overall, the dietary inclusion of SLAE in laying hen diets increased EN and EM in the current study. Our findings agree with those observed earlier studies [47]. There has never been any proof of a positive effect of dietary SLAE on EM, EN, or FCR. Adding PFA alleviated the drop in egg production as an effect of heat stress [40]. The observed improvement in EN, EM, and FCR of laying hens in the current study was probably due to the presence of some phytochemicals-plant including flavonoids, tannins, lipids, terpenes, alkaloids, steroids, and carbohydrates which were extracted from *Z. spina-christi* [20]. Moreover, the beneficial effects of dietary supplementation of plant extracts on gut health, intestinal integrity, and better utilization of nutrients have been reported, which can be linked with the improvements in the laying hen performance. In the present study, hens that supplemented with SLAE had the best FCR. These results agree with previous study [48] reporting that feeding herbal to laying birds had a positive influence on the conversion of digested feed into eggs, which is crucial for the oviposition process. We think that SLAE may have digestion-stimulating properties due to the presence of different molecules that have intrinsic effects on physiological and metabolic activities as PFA. In addition, the significant impact of PFA on laying performance could also be attributed to the positive effects of these phytogenic additives in modulating gut microbiota, enhancing nutrient digestibility and absorption, and improving ovarian characteristics resulted in better health status and subsequent laying performance [47]. So, the bioactive components in SLAE have antimicrobial and antioxidant properties, and they play a vital role in nutrient digestion and absorption [49]. The higher egg production parameters in our trial could be attributed to the antibacterial effect of SLAE, or the active ingredients of SLAE, which may increase reproductive activities, leading to increase production performance of laying hens, or to saponins present in SLAE, which leads to a direct increase in LH, and a result of the LH surge on ovulation. This may explain the increase in BW. Furthermore, it was revealed [50] evidence of a remarkable increase in the number of growing follicles, which initiated the increase in reproductive organ weight. There was an obvious increase in FSH and LH and a decrease in estradiol. Increasing FSH and LH leads to increasing the diameter of mature follicles, an effect that also may explain the increase in ovarian weight. Therefore, it could be stated that the SLAE has a positive response on production performance parameters of laying hens attributed to the presence of bioactive compounds [51]. These observations partially support the hypothesis that herbs and their extracts may favorably affect hen performance. However, the number of experimental trials with greater numbers of hens and for longer periods of time, preferably across the entire laying cycle, is still limited.

The Haugh unit is a measure of the egg’s quality inside the shell. The Haugh unit is based on the relationship between albumen height and EW (albumen quality). As compared to the control and other experimental groups, the addition of SLAE at 5.0 and 7.5 mL/kg in the laying hens’ diet resulted in the highest Haugh unit percentage [40]. Under heat stress, PFA addition in laying hen diets increased Haugh unit. In contrast to our results, [40] found no significant effect of herbal supplementation on the Haugh unit. As shown previously [52], a blend of phytogenic essential oils, including fennel oil, in laying hen diets may increase albumen quality. Improved egg quality is indicated by a greater albumen height and Haugh unit [53]. Our results also showed that dietary inclusion of SLAE influenced yolk weight during the period of the experiment, with the control group having the lowest value. The result could be linked to the heat stress that could hinder the hepatic production of vitellogenine, a protein precursor for yolk formation, as well as its release into the bloodstream [3]. Dietary SLAE may enhance the laying rate by promoting the release of vitellogenine from the liver and by increasing its concentration in the blood circulation. As a result, increases in yolk weight could be ascribed to SLAE’s antioxidant properties, which could help to maintain a healthy ovary status. In addition, eggs from hens fed herbal antioxidants supplemented diets yield eggs with increased yolk weight [54]. This influence could be explained by the increased antioxidant agents which helped hens to tolerate thermal stress during yolk formation. In the present study, the favorable effect of SLAE supplementation in laying hen diets on egg yolk color has never been documented before, and there is a scarcity of data on laying hens. According to SLAE’s antioxidant characteristics, the greater egg yolk color score in treated groups could be attributable to them [18].

Most of the criteria evaluated in this study were improved when the extract was utilised, according to data from serum metabolites and liver enzymes (Table 6). The maximum values of TP (protein is the basic composition of the egg) and their fractions occurred in treated groups. This result could be related to the maximum EW obtained with SLAE addition. The increase in serum TP concentration in treated groups can be attributed mainly to the biotin that was the major component in SLAE. Biotin is connected to major metabolic enzymes such as biotin carboxylase and biotin decarboxylase and appears to be a critical enzyme in processes such as gluconeogenesis, fatty acid synthesis, and protein synthesis [55]. In our study, the reduction in creatinine concentrations in treated birds was identical to that shown in rabbits fed SLAE powder [56].

The SLAE groups had significantly lower serum AST and ALT and within the normal range, implying improved hepatoprotective efficacy. The free radical scavenging (antioxidant) characteristics of SLAE’s components, particularly saponins and flavonoids, may be responsible for its hepatoprotective action [20]. In addition, SLAE lowered free radicals, and hence oxidative stress in animal models, as well as providing hepatic protection [57]. Tannins in *Z. spina-christi* act as detoxifying agents by limiting the development of the protein components they precipitate [36]. Thus, SLAE as phytogenic additive had beneficial effects on liver functions in heat-stressed laying hens.

Stress increases the formation of free radicals, which can damage cell membranes by triggering polyunsaturated fatty acids in the cell membrane, affecting membrane integrity [58]. In the currebt study, the level of lipid peroxidation in the blood of the treated birds could be observed as decrease in MDA concentration which was consistent with earlier research [57]. In this study, SLAE was found to have a decreasing effect on lipids, TC, and LDL while also having an augmenting effect on HDL. *Z. spina-christi* extracts from the leaves and seeds are beneficial in lowering MDA levels by lowering hyper-lipidemia, lipid peroxidation, and liver enzyme activity [59]. It has been shown that dietary SLAE inhibits lipid peroxidation and lowers the production of reactive free radicals (ROS), hence increasing the antioxidant metabolites in poultry. SLAE’s confirmed and powerful biological activities are due to the antioxidant phenolics, and flavonoids found in the leaves [18]. Metal ions are chelated, and free radicals are scavenged in these leaves, decreasing lipid peroxidation and cellular damage [59]. While hens in all SLAE treatment groups had higher activities of CAT, SOD, GSH, and TAC compared to the heat-stressed control group, the difference was significant (p<0.05), as previously mentioned [60]. Furthermore, retinoids (one of the SLAE components) have been identified to influence cell antioxidant defense mechanisms by lowering ROS and lipid peroxidation levels while enhancing SOD and GPx antioxidant enzyme activity [33], and via increasing antioxidant enzymes such as SOD and CAT [61]. The reduced MDA levels in the treated hens compared to the control group revealed that dietary SLAE can help mitigate the negative effects of heat stress by acting as an antioxidant [60]. Our findings of significantly in-creased MDA levels in heat-stressed control hens are consistent with data from heat-stressed broiler chicks [60,62] and egg-laying chickens [35]. Significantly, the activities of CAT, SOD, TAC, and GSH were higher in hens fed SLAE-supplemented diets, and this can be interpreted as a protective mechanism against oxidative stress and lipid peroxidation. This might indicate the potential of dietary SLAE to initiate the biosynthesis of antioxidant enzymes, as well as to reduce heat-stress-induced oxidative damage [63]. Interestingly, the aqueous extract of Seder leaves with a dose of 7.5 mL/kg was able to increase the activities of endogenous antioxidant enzymes (SOD, CAT, and GSH). The leaves’ aqueous extract was demonstrated to inhibit MDA of the ROS production. The mechanism that describes how dietary SLAE can reduce the negative effects of heat stress might explain that stressful environmental conditions stimulate the secretion of corticosteroids, which can be counteracted by the dietary addition of SLAE. On the other hand, recent reports suggested that flavonoids are effective antioxidants mainly because they scavenge superoxide anions [64]. This scavenging of superoxide anions is increased markedly with an increase in the concentration due to the presence of the hydroxyl group of the phenolics, which may contribute to their electron donation [65]. Similarly, Singh et al [66] showed that *Z. Spina-Christ* Fruits (ZSCF) extract might scavenge the superoxide anion in a similar way since they are rich in phenolics containing more hydroxyl groups. The inclusion of phytochemicals such as flavonoids or phenolics may explain why the ZSCF extract is a more effective scavenger of superoxide radicals. This is because phenolic compounds found in aqueous extract have a perfect structure for scavenging free radicals since they include a number of hydroxyl groups that act as hydrogen donors, making them vital and potent antioxidants [67]. All these above reasons made SLAE a good nutritional tool for reducing heat stress in layer hens, and we see some improvement in physiological characteristics.

## CONCLUSION

The current findings suggested that supplementing laying hen diets with Christ’s thorn jujube SLAE extract may improve the tolerance to heat stress-induced oxidative damage. This was demonstrated by 7.5 mL/kg SLAE dietary inclusion level which resulted in the most effective dose also to improve laying performance, egg quality and some physiological parameters of laying hens under subtropical farming conditions.

## Figures and Tables

**Table 1:** Composition and chemical analysis of the basal diet

Items	
Ingredients (%)	
Yellow corn	59.70
Soybean meal (44% CP)	24.02
Wheat bran	5.40
Corn oil	1.00
Di-Calcium phosphate	1.45
Limestone	7.77
Vitamin-mineral premix^1)^	0.30
Sodium chloride	0.30
DL-Methionine	0.06
Nutritional analysis	
Crude protein (%)	16.0
Metabolizable energy (kcal/kg)	2700
Crude fibre (%)	3.72
Calcium (%)	3.30
Available phosphorus (%)	0.40
Lysine (%)	0.90
Methionine (%)	0.35
Methionine+cysteine (%)	0.62

1) Each 3 kg of mixture contained: Vit. A, 10,000,000 IU; Vit. D_3_, 2,000,000 IU; Vit. E, 10,000 mg; Vit. K_3_, 1,000 mg; Vit. B_1_, 1,000 mg; Vit. B_2_, 5,000 mg; Vit. B_6_, 1,500 mg; Vit. B_12_, 10 mg; Pantothenic acid, 10,000 mg; Niacin, 30,000 mg; Folic acid, 1,000 mg; Biotin, 50 mg; Choline, 250,000 mg; Manganese, 60,000 mg; Zinc, 50,000 mg; Copper, 10,000 mg; Iron, 30,000; Iodine, 1,000 mg; Selenium, 100 mg; Cobalt, 100 mg; CaCO_3_ 3,000 mg.

**Table 2:** Experimental ambient temperature, relative humidity, and temperature–humidity index

Period	Ambient temperature (°C)	Relative humidity (%)	THI
wk 25 to 28	35.82±0.38	70.74±1.39	33.94±0.38
wk 29 to 32	31.92±0.64	70.23±1.19	30.13±0.65
wk 33 to 36	28.12±0.40	74.67±1.84	26.69±0.46
wk 25 to 36	32.27±0.44	71.65±0.85	30.54±0.43

THI, temperature–humidity index.

**Table 3:** Identified compounds in aqueous extract of Z*iziphus spina-christi* leaves

RT (min)	Identified compounds	Chromatogram % area
9.10	Biotin	34.66
9.46	β-Eudesmene	1.43
10.30	D-Glucuronic acid	1.81
10.40	α-Guaiene	2.11
10.42	(−)-Spathulenol	1.03
10.70	Isomyristic acid	2.29
10.98	(−)-Globulol	3.69
11.40	Farnesol	1.98
11.60	-Elemene	0.72
12.03	Hexadecane	2.2
12.40	8-cedren-13-ol	1.12
12.78	Thunbergol	2.63
13.90	Retinol	10.69
14.25	Vitexin	1.74
14.70	Linolenic acid	2.04
14.95	4′,6-Dimethoxyisoflavone-7-O-β-D-glucopyranoside	7.08
15.08	(−)-Deguelin	6.71
16.14	Trans-13-Octadecenoic acid	0.61
17.50	Nobiletin	0.96
18.86	Phytanic acid	1.59
20.98	Linalyl acetate	2.57
21.60	Cis-9-Hexadecenoic acid	1.14
21.98	Squalene	2.94
22.20	4′,7-Dimethoxy-8-methylisoflavone	3.68
23.14	Campesterol	2.58

RT, retention time.

**Table 4:** Phytochemical constituents of aqueous extract of *Ziziphus spina-christi* leaves

Phytochemical constituents	
Total flavonoids (mg of QE/100 g)^1)^	17.92±0.09
Total antioxidant capacity (mg of AC/100 g)^2)^	269.25±4.46
Saponin (%)	0.045±0.01

Values are mean±standard error (n = 5).

1) QE, quercetin equivalent.

2) AC, ascorbic acid equivalent.

**Table 5:** Effect of aqueous extract of *Ziziphus spina-christi* leaves (SLAE) addition on performance traits of dual-purpose laying hens

Parameters	Aqueous extract concentrations^1)^ (mL/kg)				SEM	p-value^2)^			
	
	Control	SLAE2.5	SLAE5	SLAE 7.5		T	L	Q	Normality
BW (g)									
Initial BW	1,316.0	1,303.4	1,319.4	1,334.7	10.25	0.111	0.0683	0.122	0.256
Final BW	1,578.0^c^	1,613.0^bc^	1,618.0^b^	1,681.5^a^	13.55	0.000	<0.001	0.294	0.864
BW gain	262.0^c^	309.6^ab^	298.6^b^	342.8^a^	12.88	0.0003	<0.001	0.895	0.164
Feed intake (g/hen/d)									
25 to 28 wk	118.3^ab^	114.5^b^	118.6^ab^	120.9^a^	1.55	0.062	0.101	0.064	0.094
29 to 32 wk	119.5^a^	115.9^b^	116.0^b^	115.2^b^	0.17	<0.001	<0.001	<0.001	0.478
33 to 36 wk	118.7^a^	113.3^c^	114.5^bc^	116.0^b^	0.50	<0.001	0.008	<0.001	0.228
25 to 36 (overall wk)	118.8^a^	114.6^c^	116.4^bc^	117.4^ab^	0.69	0.004	0.443	0.002	0.275
Feed conversion ratio (g feed/g egg)									
25 to 28 wk	10.89^b^	13.03^a^	10.53^b^	11.82^b^	0.47	0.008	0.444	0.130	0.002
29 to 32 wk	7.31^a^	6.29^b^	6.10^c^	4.92^d^	0.04	<0.001	<0.001	0.051	<0.001
33 to 36 wk	7.23^a^	6.26^b^	5.92^c^	5.43^d^	0.07	<0.001	<0.001	0.006	0.039
25 to 36 (overall wk)	8.48^a^	6.52^b^	7.34^c^	7.11^d^	0.03	<0.001	<0.001	0.540	0.598

SLAE, *Ziziphus spina-christi* leaves; SEM, standard error of means; BW, body weight.

1) SLAE2.5, diet including SLAE at 2.5 mL/kg; SLAE5, diet including SLAE at 5 mL/kg; SLAE 7.5, diet including SLAE at 7.5 mL/kg.

2) T, treatment; L, linear response; Q, quadratic response.

a–d Means within a row with various letters are significantly different (p<0.05).

**Table 6:** Effect of aqueous extract of *Ziziphus spina-christi* leaves addition on the laying performance and egg quality of dual-purpose laying hens during the experimental period

Parameters	Aqueous extract concentrations^1)^ (mL/kg diet)				SEM	p-value^2)^			
	
	Control	SLAE2.5	SLAE5	SLAE7.5		T	L	Q	Normality
Egg weight (g)									
25 to 28 wk	39.35^c^	43.05^a^	39.93^cb^	41.05^b^	0.51	<0.001	0.401	0.024	0.267
29 to 32 wk	45.48^b^	46.50^ab^	45.86^ab^	46.88^a^	0.33	0.043	0.032	0.993	0.227
33 to 36 wk	47.70^ab^	46.23^c^	47.00^bc^	48.76^a^	0.44	0.007	0.065	0.002	0.102
25 to 36 (overall wk)	44.18^b^	45.26^a^	44.26^b^	45.56^a^	0.24	0.001	0.010	0.661	0.745
Egg number (n/hen)									
25 to 28 wk	10.94^a^	8.92^b^	11.28^a^	10.88^a^	0.46	0.009	0.306	0.098	0.341
29 to 32 wk	16.42^d^	18.14^c^	19.34^b^	21.34^a^	0.28	<0.001	<0.001	0.629	0.224
33 to 36 wk	16.34^d^	18.40^c^	19.00^b^	23.40^a^	0.11	<0.001	<0.001	<0.001	0.422
25 to 36 (overall wk)	14.70^c^	15.15^c^	16.62^b^	18.62^a^	0.66	<0.001	<0.001	0.006	0.557
Egg mass (g/hen/d)									
25 to 28 wk	43.08^a^	38.43^b^	45.13^a^	44.73^a^	14.73	0.019	0.101	0.162	0.075
29 to 32 wk	74.34^d^	84.25^c^	88.95^b^	100.23^a^	14.05	<0.001	<0.001	0.545	0.436
33 to 36 wk	77.39^d^	85.78^c^	89.97^b^	114.20^a^	10.09	<0.001	<0.001	<0.001	0.106
25 to 36 (overall wk)	64.93^d^	69.47^c^	74.68^b^	86.34^a^	30.00	<0.001	<0.001	0.003	0.371
Egg quality									
Albumen diameter (mm)	62.03^b^	61.76^b^	57.80^c^	69.63^a^	1.19	<0.001	<0.001	<0.001	0.650
Albumen height (mm)	7.36^c^	7.43^c^	8.26^b^	9.53^a^	0.16	<0.001	<0.001	0.172	0.117
Albumen index (%)	11.96^b^	12.13^b^	14.53^a^	13.74^a^	0.35	0.003	0.017	0.006	0.715
Yolk diameter (mm)	36.43^ab^	35.40^b^	36.36^b^	37.50^a^	0.38	<0.001	<0.001	0.175	0.285
Yolk height (mm)	16.83^c^	17.33^b^	17.96^a^	18.00^a^	0.17	<0.001	<0.001	0.118	0.913
Yolk weight (g)	13.83^b^	13.80^b^	14.41^b^	15.04^a^	0.20	<0.001	<0.001	0.776	0.205
Yolk color	6.76^b^	8.00^a^	7.03^b^	8.36^a^	0.17	0.009	0.084	0.003	0.418
Yolk index (%)	46.41^b^	49.11^a^	49.59^a^	48.06^ab^	0.69	<0.001	<0.001	0.001	0.117
Haugh unit (score)	89.73^c^	89.68^c^	94.10^b^	99.21^a^	0.78	<0.001	<0.001	<0.001	0.861

SLAE, *Ziziphus spina-christi* leaves; SEM, standard error of means.

1) SLAE2.5, diet including SLAE at 2.5 mL/kg; SLAE5, diet including SLAE at 5 mL/kg; SLAE 7.5, diet including SLAE at 7.5 mL/kg.

2) T, treatment; L, linear response; Q, quadratic response.

a–d Means within a row with various letters are significantly different (p<0.05).

**Table 7:** Effect of aqueous extract of *Ziziphus spina-christi* leaves addition on blood biochemistry markers of heat-stressed dual-purpose laying hens

Parameters	Aqueous extract concentrations^1)^ (mL/kg)				SEM	p-value^2)^			
	
	Con	SLAE2.5	SLAE5	SLAE7.5		T	L	Q	Normality
TP (g/dL)	7.18^d^	7.52^b^	7.61^a^	7.27^c^	0.02	<0.001	<0.001	<0.001	0.178
AL (g/dL)	2.43^d^	2.55^b^	2.64^a^	2.49^c^	0.01	<0.001	<0.001	<0.001	0.238
GL (g/dL)	4.74^b^	4.97^a^	4.97^a^	4.77^b^	0.02	<0.001	0.463	<0.001	0.841
T chole (mg/dL)	115.00^a^	111.14^b^	98.71^c^	95.85^c^	1.31	<0.001	<0.001	0.707	0.557
TL (mg/dL)	452.57^a^	448.85^a^	414.85^b^	405.71^c^	1.78	<0.001	<0.001	0.142	0.394
TG (mg/dL)	225.00^a^	214.42^b^	199.00^c^	192.42^d^	1.44	<0.01	<0.001	0.179	0.109
LDL (mg/dL)	38.85^a^	32.71^b^	29.85^bc^	28.28^c^	1.10	<0.001	<0.001	0.049	0.114
HDL (mg/dL)	35.85^b^	43.28^a^	43.42^a^	46.00^a^	0.90	<0.001	<0.01	0.013	0.664
AST (U/mL)	30.71^a^	27.28^b^	27.28^b^	24.28^c^	0.88	<0.001	<0.001	0.811	0.572
ALT (U/mL)	21.57^a^	19.00^a^	14.85^b^	13.85^b^	0.91	<0.001	<0.001	0.397	0.39
Creatinine (mg/dL)	0.86^a^	0.74^b^	0.75^b^	0.75^b^	0.01	<0.001	<0.01	<0.001	0.337
Urea (mg/dL)	12.00^a^	10.14^ab^	10.71^ab^	9.00^b^	0.72	0.052	0.016	0.922	0.099

SLAE, *Ziziphus spina-christi* leaves; SEM, standard error of means; TP, total protein; AL, albumin; GL, globulin; T chole, total cholesterol; TL, total lipids; TG, triglycerides; LDL, low-density lipoprotein; HDL, high-density lipoprotein; AST, aspartate; ALT, alanine amino trans-aminases.

1) SLAE2.5: diet including SLAE at 2.5 mL/kg; SLAE5: diet including SLAE at 5 mL/kg; SLAE 7.5: diet including SLAE at 7.5 mL/kg.

2) T, treatment; L, linear response; Q, quadratic response.

a–c Means within a row with various letters are significantly different (p<0.05).

**Table 8:** Effect of aqueous extract of *Ziziphus spina-christi* leaves addition on redox status of heat-stressed dual-purpose laying hens

Parameters	Aqueous extract concentrations^1)^ (mg/kg)				SEM	p-value^2)^			
	
	Con	SLAE2.5	SLAE5	SLAE7.5		T	L	Q	Normality
TAC (mM/L)	0.092^c^	0.21^b^	0.29^a^	0.23^b^	0.01	<0.001	<0.001	<0.001	0.363
SOD (U/mL)	0.14^a^	0.16^a^	0.16^a^	0.16^a^	0.01	0.683	0.356	0.445	0.112
GSH (μM/L)	0.15^c^	0.19^b^	0.19^ab^	0.22^a^	0.01	0.007	0.001	0.650	0.079
CAT (U/mg)	0.10^b^	0.17^a^	0.16^a^	0.18^a^	0.01	<0.001	0.002	0.026	0.178
MDA (nmol/mL)	0.41^a^	0.35^b^	0.35^b^	0.32^b^	0.01	<0.001	<0.001	0.211	0.405

SLAE, *Ziziphus spina-christi* leaves; SEM, standard error of means; TAC, total antioxidant capacity; SOD, superoxide dismutase; GSH, glutathione; CAT, catalase; MDA, malondialdehyde.

1) SLAE2.5, diet including SLAE at 2.5 mL/kg; SLAE5, diet including SLAE at 5 mL/kg; SLAE 7.5, diet including SLAE at 7.5 mL/kg.

2) T, treatment; L, linear response; Q, quadratic response.

a–c Means within a row with various letters are significantly different (p<0.05).
